# Delirium Diagnosis, Complication Recognition, and Treatment Knowledge among Nurses in an Italian Local Hospital: A Cross-Sectional Study

**DOI:** 10.3390/nursrep14020059

**Published:** 2024-03-27

**Authors:** Andrea Ceccarelli, Maddalena Ballarin, Marco Montalti, Paola Ceccarelli, Silvia Mazzini, Alice Minotti, Davide Gori, Marco Senni

**Affiliations:** 1 Hygiene Unit, Department of Biomedical and Neuromotor Sciences, University of Bologna, 40126 Bologna, Italy; andrea.ceccarelli4@studio.unibo.it (A.C.);; 2 Nursing Unit, Primary Care and Community Medicine Department of Forlì-Cesena, Romagna Local Health Authority, 47521 Cesena, Italyalice.minotti@auslromagna.it (A.M.);; 3 Romagna Local Health Authority, Cesena-Valle Savio Health District, 47522 Cesena, Italy; paola.ceccarelli@auslromagna.it; 4 Nursing Unit, Primary Care and Community Medicine Department of Forlì-Cesena, Romagna Local Health Authority, 47121 Forlì, Italy; silvia.mazzini2@auslromagna.it

**Keywords:** delirium, nursing training, nursing education, nursing knowledge gaps, hospital care

## Abstract

Delirium, a multifactorial condition with an acute onset and diverse clinical manifestations, poses a significant challenge in the care of hospitalized individuals aged 65 years and older. This study aimed to evaluate the level of knowledge among nursing healthcare personnel regarding the diagnosis, recognition of complications, and treatment of delirium. A paper questionnaire consisting of 18 multiple-choice questions was distributed to nurses in twelve operational units located in four facilities within a local hospital in a specific geographical region under the jurisdiction of the Romagna Local Health Authority in Italy. Out of 194 respondents, the overall acceptance rate was 64.2%. The findings revealed an insufficient understanding of delirium among the nursing staff, with more than 40% of respondents answering incorrectly to five out of nine questions related to delirium knowledge, diagnosis, prevention, and treatment. Notably, gender emerged as a significant determinant, with female participants exhibiting a substantial odds ratio (OR) of 3.50 (*p* = 0.011 and CI95% = 1.34–9.16) compared to their male counterparts, indicating a higher likelihood of receiving delirium training among females. Furthermore, prolonged tenure within the same work context was associated with a reduced likelihood of receiving delirium training compared to those with less than two years of experience (OR = 0.21, *p* = 0.034, and CI95% = 0.05–0.89 for 6–10 years of tenure; OR = 0.22, *p* = 0.038, and CI95% = 0.05–0.92 for over 10 years of tenure). This study underscores the urgent need for enhanced delirium education and improved strategies among nurses to effectively manage patients with delirium. The results advocate regular educational sessions utilizing diverse formats to comprehensively address knowledge gaps among nursing staff. This study was not registered.

## 1. Introduction

Delirium, a multifactorial syndrome characterized by an acute onset and a spectrum of variable clinical manifestations, is generally typified by a rapid alteration in the state of consciousness, attentional deficits, and disorientation [1]. Despite commonly being associated with acute care environments, it is imperative to acknowledge that the progressive aging of the population and the resultant increase in the average age of individuals in recent years have compounded the incidence of delirium across various care settings. This burgeoning trend, coupled with the substantial morbidity and mortality associated with delirium, has transformed it into a pervasive and pertinent healthcare concern [2].

Delirium, contrary to prevalent belief, not only exhibits a higher prevalence than often perceived but also imposes a substantial health burden. Alarming statistics reveal that approximately 30 percent to 40 percent of delirium cases are preventable. Nevertheless, once it manifests, it precipitates a cascade of adverse consequences, including heightened debilitation, functional impairment, augmented morbidity and mortality rates, and escalated healthcare costs. Consequently, delirium places an onerous economic burden on healthcare facilities worldwide [3,4].

Delirium is a clinical and organizational issue involving the entire healthcare system that needs resource allocation and multidisciplinary research [5] and stands as the predominant complication observed among hospitalized individuals aged 65 years and older, impacting in excess of 2.6 million senior citizens annually within the United States [6]. Despite its notable prevalence, it frequently goes unnoticed, as demonstrated by a study by De La Cruz et al. estimating the rate of undetected delirium to be as elevated as 60% [7].

A meticulous assessment of predisposing factors and triggering elements is instrumental in identifying modifiable determinants. These factors necessitate a comprehensive, multidimensional approach, deploying targeted interventions and well-coordinated care protocols to enhance patient outcomes while mitigating potential adverse consequences such as falls, catheter-associated infections, debilitation, prolonged hospitalization, and medication mismanagement. Indeed, pre-emptive strategies that forestall the development of delirium are paramount in averting its associated complications [8].

The utmost significance of healthcare professionals, particularly those operating in hospital settings and in close proximity to hospitalized patients, cannot be overstated. Proficiency in identifying early symptoms and signs of delirium is crucial for promptly implementing preventive strategies and therapeutic interventions with the goal of averting its onset [9].

Nurses assume a crucial role in the management of delirium, given the nature of care and services they deliver. Due to their proximity to patients and vigilant clinical monitoring, nurses are uniquely positioned to discern the initial symptoms of delirium promptly and institute appropriate measures to impede its advancement. Numerous pieces of evidence substantiate the significance of nursing interventions, revealing a reduction in the incidence of delirium and associated clinical indicators through the implementation of nursing care activities [10,11].

Regarding the educational pathway for nurses, since 1990, nursing education in Italy has followed a structure comprising a three-year bachelor’s degree program, leading to professional certification. Subsequently, individuals have the option to pursue a two-year first-level master’s degree, with opportunities for further specialization through a second-level master’s degree or doctoral program. Prior to 1990, the pathway involved obtaining a specific diploma in nursing. Both diploma- and degree-holding nurses working in public hospitals are employees of the National Health Service [12].

The nursing staff employed across various hospital departments, including internal medicine, acute care, postsurgical, emergency, and palliative care, is increasingly recognizing the need for specific and updated training concerning delirium. This awareness has sparked interest in the international literature with the aim of assessing the current level of knowledge about delirium among nursing personnel before contemplating potential interventions [13,14].

Given that within Italian healthcare facilities and the international landscape previously described, the prevalence of delirium persists at a significant level, likely influenced by the advancing age of hospitalized patients and the concurrent burden of the comorbid conditions that they endure [15], the aim of this study was to examine and determine the current level of knowledge among nursing healthcare personnel pertaining to the diagnosis, recognition of complications, and treatment of elderly patients affected by delirium in a hospital setting.

## 2. Materials and Methods

During the period spanning from 5 January 2023 to 13 February 2023, paper questionnaires were administered to nurses working in 12 operational units affiliated with 4 facilities within a local hospital in a specific geographical region under the jurisdiction of the Romagna Local Health Authority in Italy. Specifically, to ensure representation from nurses in various clinical areas, the participating departments included internal medicine, emergency medicine, emergency surgery, cardiac intensive care, orthopedics, geriatrics, long-term care, short-term surgical care, intensive care, and the community hospital. Nurse coordinators of individual departments were not eligible for participation.

However, prior to the distribution of the paper questionnaires, meetings were conducted with each of the nurse coordinators from the involved departments to explain the study’s subject matter and the respective research methods. Each paper questionnaire also included an introduction outlining the study’s objectives. Participation in the study was voluntary, and the questionnaires were distributed anonymously. This research study received ethical approval from the Bioethics Committee of the University of Bologna (Italy) on 10 October 2022, under protocol number 0240051. This manuscript adheres to the Strengthening the Reporting of Observational Studies in Epidemiology (STROBE) guidelines for reporting observational research. The checklist provided by STROBE (available at https://www.equator-network.org/reporting-guidelines/strobe/, accessed on 27 March 2024) has been consulted during the preparation of this manuscript to ensure comprehensive and transparent reporting of our observational study. It is included in the Supplementary Materials for reference. There was no direct public involvement in the design, undertaking, or analysis of this research study.

### 2.1. Questionnaire

The paper questionnaire comprised 18 multiple-choice questions. The first section, consisting of the initial six questions, pertained to the socio-demographic characteristics of the participants. The subsequent section investigated the actual or perceived education related to delirium among the respondents (3 questions). Finally, the third section aimed to assess delirium diagnosis, complication recognition, and treatment knowledge among nurses. In this last section (comprising 9 questions), respondents were required to indicate the correct or false answer among four possible choices. The questions presented in the third section were formulated utilizing insights derived from the clinical expertise and collective knowledge in delirium management garnered by a multidisciplinary team comprising physicians and nurses. While the questionnaire focused on assessing fundamental knowledge regarding delirium, we opted to set a threshold of 7 out of 9 correct answers as the criterion for defining ‘adequate’ knowledge about delirium. Correct answer scores of 6 out of 9 or fewer were categorized as ‘inadequate knowledge’.

The comprehensive questionnaire translated into English is available in the Supplementary Materials (Questionnaire Instrument). Notably, none of the 18 questions mandated a compulsory response.

### 2.2. Analysis

Nurses’ responses were analyzed by computing frequency distributions for all socio-demographic and knowledge-related variables. Moreover, with respect to the multiple-choice questions regarding knowledge of certain aspects related to the prevention, diagnosis, and treatment of delirium, which required a single correct response, these were recategorized into two groups: “correct” (indicating the accurate response) and “incorrect” (encompassing all other false responses).

No formal power analysis was conducted prior to data collection. Based on resource availability and the aim of collecting enough data to provide reasonably precise estimates originating from each section of the questionnaire, including further stratified analyses, we determined a minimum size of 100 for the entire sample.

To investigate the factors contributing to the receipt of delirium-related training, a stepwise logistic regression analysis was conducted. The purpose of this analysis was to identify the variables to be included in the final multiple logistic regression model while adhering to the principles of simplicity and biological plausibility. The reference category selected was the one corresponding to the lowest level or class. The results of the multivariate analyses were presented as odds ratios (ORs) along with their corresponding 95% confidence intervals (95% CIs). Statistical significance was defined as *p* < 0.05. To assess the robustness of the collected sample, we conducted a complete case analysis followed by a sensitivity analysis, comparing it with the entire group. Our findings revealed no significant differences or measurable biases in our sample that could be attributed to missing data.

Data collection was conducted using Microsoft Excel (2018), while all statistical analyses were performed using Stata 15 software (StataCorp, College Station, TX, USA).

## 3. Results

### 3.1. Main Features of the Sample

In general, a total of 194 nurses, out of the 302 invited, responded to the e-questionnaire, resulting in an overall acceptance rate of 64.2%. Table 1 provides a comprehensive overview of the primary socio-demographic characteristics of the study sample. Notably, the distribution of participant ages revealed a diverse demographic landscape, with 41.0% falling within the 26–35 age group, indicating this to be the predominant cohort, followed by 21.8% in the 36–45 age range. Regarding gender, the study sample was predominantly composed of females, constituting 86.2% of the total respondents. Concerning departmental affiliation, a significant proportion of participants were associated with the medicine department (37.1%), closely followed by those in intensive care (35.1%), and with a smaller proportion in surgery (24.2%) and community hospital (3.6%). An exploration of their professional tenure revealed a diversified distribution, with 50.0% having accumulated over a decade of experience in their respective professions, while 23.7% had from 2 to 5 years of professional experience. Additionally, participants’ educational background was examined, indicating that 62.9% held a bachelor’s degree, 19.1% possessed a high school diploma, and 18.0% had pursued postgraduate education. Furthermore, the table sheds light on respondents’ self-perceived understanding of delirium and its associated prevention and treatment, with 68.9% considering their knowledge satisfactory, and 24.9% characterizing it as inadequate. In conclusion, the data revealed that 60.5% of respondents had undergone specialized training in delirium prevention and treatment strategies, while 39.5% had not participated in such instructional programs.

### 3.2. Knowledge of Delirium Diagnosis, Prevention, and Treatment

Table 2 presents the distribution of correct and incorrect knowledge among participants regarding delirium and its associated prevention and treatment. The data indicate that most respondents had correct knowledge regarding the definition of delirium (56.2%), the settings where it can occur (85.6%), and the first-line therapy for hypoactive and hypokinetic forms of delirium (57.0%). However, 91% demonstrated an incorrect understanding of the first-line therapy for hyperactive and hyperkinetic forms of delirium, as well as the identification of contextual factors that can serve as delirium triggers (85.0%). An overwhelming consensus was observed on the role of a geriatrician-led team in mitigating acute delirium problems (98.9%) and the nurse’s role in delirium prevention (97.4%). Conversely, a minority of participants incorrectly believed that family members should not leave during an acute delirium episode (19.2%).

Analyzing the responses provided by individual participants to the questions pertaining to delirium knowledge, we calculated the total number of correct answers, which ranged from one to nine, for each respondent. The majority of respondents (*n* = 138, 71.1%) scored within the range from four to six correct answers out of the nine available, while only 41 individuals (21.3%) reached the threshold set for adequate knowledge. Specifically, 5 (2.6%) participants achieved a total score of two, 10 (5.2%) participants scored three correct answers, 34 (17.5%) participants scored four, 51 (26.3%) participants scored five, 53 (27.3%) participants scored six, 35 (18.0%) participants scored seven, and 6 participants scored eight correct answers (3.1%).

### 3.3. Multivariate Analysis

The multivariate analysis findings, as summarized in Table 3, present odds ratios (ORs) and their corresponding 95% confidence intervals (CIs) for various variables pertinent to the reception of delirium training. The outcomes of the stepwise regression analysis revealed significant associations with gender and time in the current work context (*p* < 0.05). In addition to these variables, age, department affiliation, time practicing the profession, and educational attainment were also integrated into the model for the sake of plausibility. To enhance clarity, statistically significant associations are conspicuously highlighted.

Age, upon thorough examination, displayed no consistent and statistically significant association with the delivery of delirium training. However, gender emerged as a noteworthy determinant, revealing that female participants exhibited a substantial OR of 3.50 (*p* = 0.011 and CI95% = 1.34–9.16) compared to their male counterparts, signifying a heightened propensity for receiving delirium training among the former. Notably, the duration spent in the current work context exhibited significant associations with delirium training. Specifically, a prolonged tenure within the same work context was found to diminish the likelihood of receiving training on delirium when compared to those with less than two years of experience in the current context (OR = 0.21, *p* = 0.034, and CI95% = 0.05–0.89 for individuals working in the same context for 6–10 years; OR = 0.22, *p* = 0.038, and CI95% = 0.05–0.92 for those with over 10 years of tenure). Conversely, department-based variations did not achieve statistical significance, mirroring the lack of statistically significant associations for years of practice and educational level.

## 4. Discussion

In this study, the knowledge of delirium among nursing staff in an Italian hospital was assessed. The results highlighted the heterogeneous nature of knowledge within our sample, with no significant association with socio-demographic variables. Overall, the knowledge level was found to be inadequately aligned with the increasing clinical impact of delirium on healthcare services [5,15]. Similar levels of poor delirium knowledge have been reported in previous studies in China and the Netherlands [14,16].

This study was well-received by professionals, achieving a high acceptance rate. Regarding the sample characteristics, the majority consisted of young and female participants. On average, Italian nurses tend to be older [17]. However, the age distribution is unsurprising, considering the current occupational landscape in the Italian healthcare sector. This pattern is associated with the practice of deploying younger professionals in hospitals, where acute pathologies are treated, while older professionals are more commonly assigned to outpatient facilities or roles focused on chronic and community care. The prevalence of the female gender not only aligned with regional and national contexts [18] but was also consistent with international studies [19]. This suggests the influence of cultural factors promoting gender diversity in nursing, warranting future analyses of factors contributing to the overall attractiveness of the nursing profession for women.

From a socio-demographic perspective, examining professional seniority revealed that one-third of respondents had been working for more than 10 years, while sixty percent had been in their current position for less than 5 years. These figures were attributed to the widespread job rotation policy in the Italian hospital nursing context, which the literature indicates could enhance job satisfaction and commitment among nurses [20]. A Jordanian study corroborated this, demonstrating elevated levels of job satisfaction and job commitment among nurses who experienced job rotation [21]. The intense nursing turnover during the COVID-19 pandemic may have also contributed to these trends [22].

When respondents were asked to indicate their perceived knowledge of delirium, 69% of participants considered themselves adequately prepared for delirium prevention and treatment. However, upon analysis of respondents’ answers to questions regarding the diagnosis, prevention, and treatment of delirium, it was evident that the majority of the sample lacked adequate knowledge. Significant gaps were identified, for instance, in knowledge of first-line therapy for hyperactive or hyperkinetic delirium (91% incorrect responses), the identification of contextual factors as triggers for delirium (85% incorrect responses), and the use of diagnostic tools for delirium (53% incorrect responses). This discrepancy may be attributed to the phenomenon of ‘self-perceived knowledge’, where existing knowledge influences further information acquisition. Other similar phenomena are observed in healthcare contexts, as evidenced by Scheinberg-Andrews et al. who investigated the ‘self-perceived knowledge’ of oncology nurses regarding end-of-life knowledge [23] and Hou et al. who indicated inadequate clinical management of delirium despite moderate ‘self-perceived knowledge’ among nurses [24].

These findings prompt reflection on strategies for targeted continuing education aimed at improving nurses’ knowledge and practice behaviors related to delirium. This consideration is especially relevant given that, as indicated, a significant proportion of respondents reported not having received any training on delirium at all.

In the context of delirium training, gender has emerged as a significant determinant, wherein females exhibit a notable odds ratio for undergoing delirium training. However, the limited number of male nurses operating within the hospital and participating in the study diminishes the predictive power of the logistic regression. Prolonged tenure in the same work context reduced the likelihood of receiving delirium training significantly. This observation suggested that the policy of ‘job rotation’ between hospital departments may enhance the likelihood of receiving delirium training, as also highlighted by Jordan et al. in their work [25]. Notably, no discernible differences were found between educational backgrounds or age groups, emphasizing the need for targeted continuing education for all demographics.

Our study’s findings align with the existing literature, indicating insufficient delirium knowledge among nursing staff. For example, a study in the USA revealed low recognition rates for different subtypes of delirium among licensed nurses and nursing assistants [26]. Similarly, a study by Papaioannou et al. found that nurses in departments with a higher incidence of delirium often answered delirium-oriented questions incorrectly [14].

In light of our results and those of other scientific research, there is a need for the development of targeted delirium-related training programs to address this crucial knowledge gap at the local level. Some studies have demonstrated the effectiveness of educational interventions aimed at improving nurses’ knowledge and practice of delirium assessment, using standard educational programs or specific tools such as The Confusion Assessment Method for the Intensive Care Unit [27,28]. These interventions could be adapted to other contexts where the knowledge gap on delirium among nurses needs to be addressed.

### Limitations

This study has several limitations. Firstly, the convenience sampling method employed for participant selection deviates from the ideal representative sampling approach. Despite a notably high voluntary participation rate, the potential for selection bias persists due to participants’ fear of consequences stemming from a lack of knowledge on the subject. To address this bias, we deliberately excluded the coordinating staff of the various departments involved in this study. Additionally, we proactively assured participants that their data would be anonymized and presented in an aggregated form. In an effort to further mitigate selection biases, especially those associated with digital literacy often linked to age, the administration of a paper-based questionnaire was chosen for study participation. However, it is noteworthy that the majority of respondents were young, aligning with the current employment landscape of nurses in the Italian context, where younger professionals are typically employed in hospital settings dealing with acute conditions, while older professionals are engaged in community and chronic care. The utilization of a paper-based questionnaire introduced an additional limitation as respondents were not obligated to answer all questions, resulting in varying denominators for each item in the final analysis. While we acknowledge the importance of community engagement, constraints such as time, resources, and study design limited our ability to actively involve the public in the research process. Despite this limitation, we remain committed to transparency and the responsible reporting of our findings. We recognize the potential impact of public involvement and aim to integrate community perspectives in future research endeavors to enhance the relevance and applicability of our work. Finally, it is essential to recognize the inherent limitations associated with the logistic regression analysis utilized in this study. A significant constraint stems from the relatively small sample size employed in this investigation. The restricted number of observations elevate the risk of overfitting, whereby the model may capture noise rather than genuine relationships between predictors and the outcome variable. Moreover, the estimates of coefficients may lack precision, potentially impeding the detection of significant effects and diminishing predictive accuracy. Despite these limitations, the findings of this study offer valuable insights within the scope of available data, emphasizing the need for cautious interpretation and further validation in future research endeavors. Given the precautions taken, we believe our study provides a reliable and valuable snapshot of the current knowledge regarding delirium among nursing staff within an Italian LHA.

## 5. Conclusions

In conclusion, our study sheds light on the varied and insufficient knowledge on delirium among nursing staff in an Italian hospital, revealing a lack of alignment with the growing clinical impact of delirium on healthcare services. Despite self-perceived adequacy in delirium preparedness, our findings uncovered specific knowledge gaps, particularly in areas crucial for effective delirium prevention and treatment. The identified factors influencing training receipt, such as gender and job rotation policies, underscore the importance of targeted continued education for all demographics. Importantly, our results resonate with the global literature, emphasizing the urgent need for focused delirium-related training programs to bridge the existing knowledge gap among nursing staff.

## Figures and Tables

**Table 1:** The main characteristics of the study sample (*n* = 194). For each characteristic, the total number of responses are provided, as responses to the questionnaire were not mandatory.

Characteristics		*n* (%)
Age (years) (*n* = 188)	20–25	16 (8.5)
	26–35	77 (41.0)
	36–45	41 (21.8)
	46–55	40 (21.3)
	>55	14 (7.5)
Gender (*n* = 189)	Male	26 (13.8)
	Female	163 (86.2)
Department (*n* = 194)	Medicine	72 (37.1)
	Surgery	47 (24.2)
	Intensive Care	68 (35.1)
	Community Hospital	7 (3.6)
Time practicing profession (years) (*n* = 194)	<2	11 (5.7)
	from 2 to 5	46 (23.7)
	from 6 to 10	40 (20.6)
	>10	97 (50.0)
Time in current work context (*n* = 194)	<2	55 (28.4)
	from 2 to 5	59 (30.4)
	from 6 to 10	18 (9.3)
	>10	62 (32.0)
Education level (*n* = 194)	High school diploma	37 (19.1)
	Bachelor’s degree	122 (62.9)
	Further education	35 (18.0)
Self-perception of knowledge on delirium and related prevention and treatment (*n* = 193)	Inadequate	48 (24.9)
	Satisfactory	133 (68.9)
	Excellent	12 (6.2)
Specific training on delirium prevention and treatment strategies (*n* = 190)	No	75 (39.5)
	Yes	115 (60.5)

**Table 2:** Knowledge of delirium and related diagnosis, prevention, and treatment. For each question, the total number of responses are provided, as responses to the questionnaire were not mandatory.

	Correct Knowledge	Incorrect Knowledge
Definition of delirium (*n* = 185)	104 (56.2)	81 (43.8)
Setting where delirium can occur (*n* = 194)	166 (85.6)	28 (14.4)
Utilization of delirium diagnostic tool (*n* = 183)	86 (47.0)	97 (53.0)
First-line therapy for hypoactive or hypokinetic delirium (*n* = 193)	110 (57.0)	83 (43.0)
First-line therapy for hyperactive or hyperkinetic delirium (*n* = 189)	17 (9.0)	172 (91.0)
Identification of contextual factors that can be delirium triggers (*n* = 193)	29 (15.0)	164 (85.0)
The role of a geriatrician-led team in mitigating acute delirium problems (*n* = 190)	188 (98.9)	2 (1.1)
Nurse’s role in delirium prevention (*n* = 191)	186 (97.4)	5 (2.6)
Family members should leave during an acute delirium episode (*n* = 193)	156 (80.3)	37 (19.2)

**Table 3:** Multivariate analysis identifying factors associated with delirium training. Each category is presented alongside its absolute frequency (Statistically significant *p*-Value are indicated in bold).

Training Received on Delirium		OR	SE	*p*-Value	CI95%
Age (years)	20–25 (*n* = 16)	1			
	26–35 (*n* = 77)	0.50	0.40	0.390	0.10–2.42
	36–45 (*n* = 41)	0.50	0.50	0.491	0.07–3.64
	46–55 (*n* = 40)	0.75	0.80	0.786	0.09–6.09
	>55 (*n* = 14)	0.32	0.40	0.363	0.03–3.71
Gender	Male (*n* = 26)	1			
	Female (*n* = 113)	3.50	1.72	**0.011**	1.34–9.16
Department	Medicine (*n* = 72)	1			
	Surgery (*n* = 43)	0.69	0.30	0.401	0.29–1.64
	Intensive Care (*n* = 68)	0.48	0.20	0.078	0.21–1.09
	Community Hospital (*n* = 2)	3.12	3.73	0.342	0.30–32.5
Time practicing profession (years)	<2 (*n* = 11)	1			
	from 2 to 5 (*n* = 46)	1.27	1.12	0.221	0.22–7.23
	from 6 to 10 (*n* = 40)	2.15	2.16	0.300	0.30–15.36
	>10 (*n* = 97)	2.22	2.50	0.244	0.24–20.22
Time in current work context	<2 (*n*= 55)	1			
	from 2 to 5 (*n* = 59)	0.90	0.45	0.839	0.34–2.40
	from 6 to 10 (*n* = 18)	0.21	0.16	**0.034**	0.05–0.89
	>10 (*n* = 62)	0.22	0.16	**0.038**	0.05–0.92
Education level	High school diploma (*n* = 37)	1			
	Bachelor’s degree (*n* = 122)	0.38	0.26	0.150	0.10–1.42
	Further education (*n* = 35)	1.23	0.90	0.776	0.29–5.17

## Data Availability

The data presented in this study are available on request from the corresponding author.

## Supplementary Materials

The following supporting information can be downloaded at: https://www.mdpi.com/article/10.3390/nursrep14020059/s1, STROBE checklist; Questionnaire Instrument.

## Public Involvement Statement

There was no public involvement in any aspect of this research.

## Guidelines and Standards Statement

This manuscript adheres to the Strengthening the Reporting of Observational Studies in Epidemiology (STROBE) guidelines for reporting observational research. The checklist provided by STROBE (available at https://www.equator-network.org/reporting-guidelines/strobe/, accessed on 27 March 2024) has been consulted during the preparation of this manuscript to ensure comprehensive and transparent reporting of our observational study. The checklist is included in the Supplementary Materials for reference.

## References

[1] Sachdev P.S., Blacker D., Blazer D.G., Ganguli M., Jeste D.V., Paulsen J.S., Petersen R.C. (2014). Classifying Neurocognitive Disorders: The DSM-5 Approach. Nat. Rev. Neurol..

[2] Gao W., Zhang Y., Jin J. (2021). Poor Outcomes of Delirium in the Intensive Care Units Are Amplified by Increasing Age: A Retrospective Cohort Study. World J. Emerg. Med..

[3] Setters B., Solberg L.M. (2017). Delirium. Prim. Care Clin. Off. Pract..

[4] Igarashi M., Okuyama K., Ueda N., Sano H., Takahashi K., Qureshi Z.P., Tokita S., Ogawa A., Okumura Y., Okuda S. (2022). Incremental medical cost of delirium in elderly patients with cognitive impairment: Analysis of a nationwide administrative database in Japan. BMJ Open.

[5] Frisardi V., Nicolini M., Cautero N., Ghirardelli R., Davolio F., Haouili M., Barani M. (2022). Proposing a Scientific and Technological Approach to the Summaries of Clinical Issues of Inpatient Elderly with Delirium: A Viewpoint. Healthcare.

[6] Inouye S.K., Westendorp R.G., Saczynski J.S. (2014). Delirium in Elderly People. Lancet.

[7] De La Cruz M., Fan J., Yennu S., Tanco K., Shin S., Wu J., Liu D., Bruera E. (2015). The Frequency of Missed Delirium in Patients Referred to Palliative Care in a Comprehensive Cancer Center. Support Care Cancer.

[8] Hshieh T.T., Inouye S.K., Oh E.S. (2018). Delirium in the Elderly. Psychiatr. Clin. N. Am..

[9] Lange S., Mȩdrzycka-Dabrowska W., Tomaszek L., Wujtewicz M., Krupa S. (2023). Nurses’ Knowledge, Barriers and Practice in the Care of Patients with Delirium in the Intensive Care Unit in Poland—A Cross-Sectional Study. Front. Public Health.

[10] Tan C.M., Camargo M., Miller F., Ross K., Maximous R., Yung P., Marshall C., Fleming D., Law M., Tsang J.L. (2019). Impact of a Nurse Engagement Intervention on Pain, Agitation and Delirium Assessment in a Community Intensive Care Unit. BMJ Open Qual..

[11] Schubert M., Bettex D., Steiger P., Schrch R., Haller A., Bogdanovic J., Garcia Nuez D., Schwarz U., Siegemund M. (2020). Implementation of a Multiprofessional, Multicomponent Delirium Management Guideline in Two Intensive Care Units, and Its Effect on Patient Outcomes and Nurse Workload: A Pre-Post Design Retrospective Cohort Study. Swiss. Med. Wkly..

[12] Gazzetta Ufficiale. https://www.gazzettaufficiale.it/eli/id/1995/01/09/095G0001/sg.

[13] Morandi A., Di Santo S.G., Zambon A., Mazzone A., Cherubini A., Mossello E., Bo M., Marengoni A., Bianchetti A., Cappa S. (2019). Delirium, Dementia, and In-Hospital Mortality: The Results from the Italian Delirium Day 2016, A National Multicenter Study. J. Gerontol. Ser. A.

[14] Papaioannou M., Papastavrou E., Kouta C., Tsangari H., Merkouris A. (2023). Investigating Nurses’ Knowledge and Attitudes about Delirium in Older Persons: A Cross-Sectional Study. BMC Nurs..

[15] Yuan Y., Lei B., Li Z., Wang X., Zhao H., Gao M., Xue Y., Zhang W., Xiao R., Meng X. (2022). A Cross-Sectional Survey on the Clinical Management of Emergence Delirium in Adults: Knowledge, Attitudes, and Practice in Mainland China. Brain Sci..

[16] Van Velthuijsen E.L., Zwakhalen S.M.G., Mulder W.J., Verhey F.R.J., Kempen G.I.J.M. (2018). Detection and Management of Hyperactive and Hypoactive Delirium in Older Patients during Hospitalization: A Retrospective Cohort Study Evaluating Daily Practice. Int. J. Geriatr. Psychiatry.

[17] National Agency for Regional Health Services (2022). Il Personale del Servizio Sanitario Nazionale. https://www.agenas.gov.it/images/agenas/In%20primo%20piano/personale/personale_ssn_2022.pdf.

[18] Italian Ministry of Health Personale delle ASL e Degli Istituti di Ricovero Pubblici ed Equiparati–Anno 2021. https://www.salute.gov.it/portale/documentazione/p6_2_2_1.jsp?id=3348.

[19] Lu H., Hou L., Zhou W., Shen L., Jin S., Wang M., Shang S., Cong X., Jin X., Dou D. (2021). Trends, Composition and Distribution of Nurse Workforce in China: A Secondary Analysis of National Data from 2003 to 2018. BMJ Open.

[20] Platis C., Ilonidou C., Stergiannis P., Ganas A., Intas G., Vlamos P. (2021). The Job Rotation of Nursing Staff and Its Effects on Nurses’ Satisfaction and Occupational Engagement. GeNeDis 2020.

[21] Alfuqaha O.A., Al-Hairy S.S., Al-Hemsi H.A., Sabbah A.A., Faraj K.N., Assaf E.M. (2021). Job Rotation Approach in Nursing Profession. Scand. Caring Sci..

[22] Tolksdorf K.H., Tischler U., Heinrichs K. (2022). Correlates of Turnover Intention among Nursing Staff in the COVID-19 Pandemic: A Systematic Review. BMC Nurs..

[23] Scheinberg-Andrews C., Ganz F. (2020). Israeli Nurses’ Palliative Care Knowledge, Attitudes, Behaviors, and Practices. ONF.

[24] Hou X., Li X., Guo R., Wang Y., He S., Yang H., Bai D., Lu Y. (2023). Knowledge and Practice Behaviors Toward the Care of the Dying Among Chinese Oncology Nurses: A Cross-Sectional Survey. J. Hosp. Palliat. Nurs..

[25] Jordan S., Brauner E. (2008). Rotation in der Anästhesiepflege—Eine Analyse der Wirkungen auf Wissens- und Lernprozesse. Pflege.

[26] Steis M.R., Behrens L., Colancecco E.M., Mogle J., Mulhall P.M., Hill N.L., Fick D.M., Kolankowski A.M. (2015). Licensed Nurse and Nursing Assistant Recognition of Delirium in Nursing Home Residents With Dementia. Ann. Long-Term Care.

[27] Aldawood Z.S., Alameri R.A., Elghoneimy Y., Swyan A.H.A., Almulla H., Hammad S.S., Saleh N.S.A., Alameri S.A. (2023). Impact of Educational Program on Critical Care Nurses’ Knowledge of ICU Delirium: A Quasi-Experimental Study. Med. Arch..

[28] Baluku Murungi E., Niyonzima V., Atuhaire E., Nantume S., Beebwa E. (2023). Improving Nurses Knowledge and Practices of Delirium Assessment at Mbarara Regional Referral Hospital: A Quasi Experimental Study. Adv. Med. Educ. Pract..

