# Supplementary material for: Delirium Diagnosis, Complication Recognition, and Treatment Knowledge among Nurses in an Italian Local Hospital: A Cross-Sectional Study
Source: Nurs Rep. 2024 Mar 27;14(2):767–76. doi: 10.3390/nursrep14020059 (PMC11036222; doi:10.3390/nursrep14020059)
Supplement: Supplementary file 1 [file nursrep-14-00059-s001.zip › nursrep-2798118-supplementary.pdf]

Questionnaire Instrument

1) Age: ____ (in years)

2) Gender: □ M □ F

3) Operational unit (O.U.) in which you are currently working:
- Medical area
- Surgical area
- Critical care area
- Elderly residential facilities
- OSCO (territorial healthcare hospitalization unit)
- Other (specify)_______

4) Period of time since which you have been practicing: _________________________

5) Period of time in the current O.U./work context in which you are now working:
____

6) Educational qualification obtained:
- High school diploma
- Bachelor's degree
- Further Education

7) Perception regarding one's knowledge about Delirium and related behaviors to adopt for its prevention and treatment:
- Excellent
- Satisfactory
- Inadequate

8) Specific training received on delirium prevention and treatment strategies.
- Yes
- No

9) If you answered Yes to the previous question, by which modalities:
- Self-training
- During basic training
- Through participation in courses/conferences on voluntary participation

10) In your opinion, which of the following definitions is most suitable to describe the definition of delirium?
□ Psychotic-like behavioral disorder that often accompanies the natural history of dementia disease
□ Cognitive disorder (acute spatial and/or temporal disorientation with relative memory impairment)
□ Acute or subacute cognitive disorder with a tendency to fluctuate, with an inability to maintain and shift attention

□ Behavioral disorder not otherwise characterized, typical of atypical forms of dementia (e.g., DLB)

11) In your opinion, delirium disorder is an issue in which setting:
□ Geriatrics and long-term care
□ Predominantly in RSAs
□ All hospital wards
□ Only in surgical wards

12) Which diagnostic tool do you use for the diagnosis of delirium?
□ None
□ Neecham
□ CAM
□ Other

13) What is the first approach you would take for the treatment of hypoactive or hypokinetic delirium:
□ None
□ Pharmacological
□ Non-pharmacological (search for causes and behavioral approach)
□ Pharmacological and non-pharmacological

14) What is the first approach you would take for the treatment of hyperactive or hyperkinetic delirium:
□ None
□ Pharmacological
□ Non-pharmacological (search for causes and behavioral approach)
□ Pharmacological and non-pharmacological

15) In your opinion, which of the following should be considered and treated as a possible contextual factor of delirium? (Choose as many options as you consider correct)
□ Polytherapy
□ Infections
□ Water balance alterations
□ Urinary retention
□ Bowel obstruction
□ Bladder catheter
□ Sleep disorders
□ Malnutrition

16) How do you evaluate this statement: "an intervention program based on the management of patients by a multidisciplinary team coordinated by a geriatrician reduces the incidence of delirium in elderly patients hospitalized for acute medical problems"?
□ True, I agree
□ True, but only for certain types of patients
□ False, I do not believe that the involvement of a multidisciplinary team is necessary
□ False, the intervention of the geriatrician is sufficient.

17) In your experience, can the nurse contribute to the prevention of delirium?
□ No
□ No, delirium cannot be prevented
□ Yes, by administering the prescribed therapy
□ Yes, by identifying the predisposing factors (advanced age, cognitive deficit, sensory deficit, presence of severe pathologies, alcoholism, malnutrition...) and preventing the precipitating factors (sleep deprivation, dehydration, immobility, etc.)

18) Which of these statements do you think is false?
□ The family can become a resource and a considerable support for the patient in case of delirium
□ The family and the patient must be educated about delirium, its causes, and its course
□ During delirium episodes family members should leave the patient as they may be a triggering factor
□ The nurse can contribute to the prevention of delirium episodes
